# Lane Departure Assessment via Enhanced Single Lane-Marking

**DOI:** 10.3390/s22052024

**Published:** 2022-03-04

**Authors:** Yiwei Luo, Ping Li, Gang Shi, Zuowei Liang, Lei Chen, Fengwei An

**Affiliations:** 1 School of Microelectronics, Southern University of Science and Technology, Shenzhen 518055, China; 12132463@mail.sustech.edu.cn (Y.L.); 12032805@mail.sustech.edu.cn (G.S.); 11930177@mail.sustech.edu.cn (Z.L.); 2 Department of Computing, The Hong Kong Polytechnic University, Hong Kong 999077, China; p.li@polyu.edu.hk

**Keywords:** lane departure warning, 3D imaging model, extrinsic camera parameters, lane departure assessment

## Abstract

Vision-based Lane departure warning system (LDWS) has been widely used in modern vehicles to improve drivability and safety. In this paper, a novel LDWS with precise positioning is proposed. Calibration strategy is first presented through a 3D camera imaging model with only three parallel and equally spaced lines, where the three angles of rotation for the transformation from the camera coordinate system to the world coordinate system are deduced. Then camera height is calculated compared to the previous works using a measured one with potential errors. A criterion for lane departure warning with only one of the two lane-markings is proposed to estimate both yaw angle and distance between the lane-markings and the vehicle. Experiments show that calibration strategy can be easily set up and achieve an average of 98.95% accuracy on the lane departure assessment.

## 1. Introduction

Mobility plays an important role in modern society, and it provides a high quality life for humans. However, according to WHO, tens of millions of people are injured or disabled because of road accidents [1], making the Safety Driving Assist System (SDAS) necessary to protect drivers’ safety. An example of research in SDAS is a model constructed by Wang et al. [2] based on the host–target vehicle dynamics and road constraints to estimate the lateral motion of the preceding target vehicles. A complete system proposed by Lin et al. consists of lane change detection, forward collision warning, and overtaking vehicle identification [3]. As an important part of SDAS, the lane departure warning system (LDWS) is designed to warn the drivers when the vehicles tend to deviate from their lanes, effectively preventing traffic accidents which are mostly out of driver’s inattention or lack of experience.

### 1.1. Related Work

Over the years, LDWS is still attractive in its decision-making algorithms. Martínez-García et al. [4] characterized a concept of elementary steering pulses through machine learning to model human lane keeping control. Zhang et al. [5] proposed a lane departure warning algorithm based on probability statistics of driving habits to make lane departure warnings more targeted and accurate. Chen et al. [6] proposed a human-machine shared control strategy based on hybrid system theory, and the results showed good human–machine coordination.

Moreover, some researchers focused their works on evaluating the safety level of LDWS. One of the typical research in [7] presented an experimental test to show the main predictors of system fault. Another research in [8] identified the characteristics of lane departure crashes and quantified the safety potential of LDWS.

Among all the LDWS algorithms, vision-based algorithms play an important role, which can be divided into two types: (1) algorithms using only image information and (2) algorithms using image information and road model. The first type determines lane departure warning only by using the information in the image, e.g., the slopes of the lane markings in the image can estimate the vehicle’s turning direction. This type attracts many researchers because of its simplicity, while one of its shortcomings is the lack of robustness. An edge distribution function (EDF) in [9] determined lane departure using the position of the symmetry axis of EDF. However, the camera setup should be ideal enough; otherwise, the symmetry axis of EDF may change even if lane departure is not happening. Vijay et al. [10] presented a similar algorithm using the deviation of the centroid line of detected lanes from the center of the image. A lane departure identification method used three lane-related parameters, including the Euclidean distances between every two points of the Hough origin H_o_, the midpoints mp_1_ and mp_2_ of the identified left and right lane-markings to identify the state of departure [11,12,13]. Besides, algorithms judging the (*ρ*, *θ*) patterns or just one of the detected left, and right lane-markings determined the left or right lane departure situation [14,15,16,17,18,19,20,21,22,23,24]. The recent study conducted by Lin et al. determines lane departure also by the information of the detected lane-markings only, and it uses a state machine to recognize the “left,” “right,” and “normal” status, which can reduce the false alarms when the lane-marking is blocked by obstacles [3].

The vision-based type usually involves the camera calibration to transform the image into the real world. Xu et al. [25] proposed a camera calibration method with a set of lines parallel and perpendicular to the ground plane to determine the camera parameters, including the camera’s three deviation angles and focal length. Then the distance between the car and road boundaries was obtained using the pre-measured camera height. A mapping algorithm between the image and road coordinates remapped the detected lanes to the actual roads, but only deviation angles on two dimensions of the camera (i.e., α_0_ and β_0_) were considered [26]. In [27], the ratio of the slopes of the detected left and right lanes represented the degree of lateral offset of the vehicle, and the slopes were calculated using the relation between the world and camera coordinates. However, only one of all three deviation angles of the camera (i.e., pitch angle) is considered nonzero. The algorithm based on probability statistics of driving habits described in [5] also uses the matrix transformation of the image coordinate system and the world coordinate system, but no deviation angle of the camera is mentioned in the transformation.

Besides, vision-based lane departure warning algorithms are similar to vision-based vehicle localization methods like Simultaneous Localization And Mapping (SLAM) since both aim to get the vehicle’s position relative to the environment. Lin et al. proposed a vehicle localization method based on topological map construction and scene recognition [28]. The Omni-directional image sequences construct the topological map in this work. The proposed method collects multiple feature points in the input images and can output vehicle position and recognition of scenes.

### 1.2. Contributions

**Figure 1:**
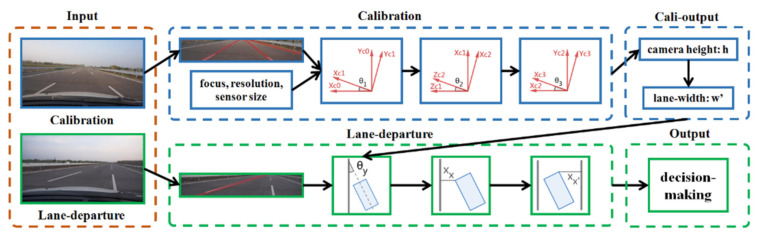
Overview of our proposed method. First, calibration with three parallel and equally spaced lines on the ground is applied to estimate the three angles of rotation for the transformation from the camera coordinate system to the world coordinate system. Next, camera height is calculated, then lane-width can be calculated from the camera height. After calibration, lane departure warning is done by estimating the distance between the lane-marking and the vehicle as well as the yaw angle of the vehicle to the lane with only one of the two lane-markings. Finally, decision is made using the estimated distance and angle.

In this paper, we propose a new algorithm of LDWS of the second type, which involves easy calibration and warning processes with only one of the two-lane markings. Precisely, the front camera of the vehicle is calibrated to obtain the relationship between the image and the real world. The lane detection technique is used to detect only one lane-marking in each image. This proposed lane departure warning method, combining the “only one” lane-marking information and the calibration information, can determine whether the vehicle has the potential to deviate from the lane. Figure 1 shows an overview of the proposed method. On the contrary to the previous works, this method only requires the information of one lane-marking in the image and only outputs vehicle direction and distance relative to the detected lane-marking.

In [5,26,27], the deviation angles on all three dimensions were not taken into account. This makes the algorithms less reliable since deviation angles of a camera affect the mapping accuracy or vehicle positioning. In [25], its calibration process required two horizontal lines and two vertical lines, which means there should be objects perpendicular to the ground in the environment. However, the calibration process of our proposed algorithm requires the environment to contain three parallel and equally spaced horizontal lines, which is much easier to satisfy. Neither [25], nor our proposed method utilizes the existing and mature camera calibration methods. The reason is that the existing methods always need a calibration object with a given shape and size like a chessboard, and they require particular calibration apparatuses and an elaborate setup. Besides, camera parameters like deviation angles are subject to environmental change. Therefore, the camera should be repeatedly calibrated in the case of the mature calibration method. In addition, in [25], the camera height is manually measured, while in our proposed method, the camera height can be calculated during the calibration processing without measured errors. To sum up, the contributions of this work are summarized as follows:

1. A calibration strategy with only three parallel and equally spaced lines is applied to estimate the three rotation angles to transform the camera coordinate system to the world coordinate system through a 3D imaging model. Compared to the method in [25], this model with no vertical lines enables the camera can be equipped in the front of the car without dedicated angles;
2. The camera height and lane-width can be calculated instead of measured, using estimated camera extrinsic parameters in the proposed calibration strategy. This method avoids errors during the measurement;
3. A criterion for lane departure warning is proposed by estimating the yaw angle and distance between the lane markings and the vehicle with only one of the two lane markings. This criterion is simple and reliable compared to the traditional algorithms, which should detect both lane-markings.

## 2. Camera Calibration

The direct relation between calibration and lane departure warning is that camera calibration transforms the image coordinate into the real-world coordinate, and a mapping algorithm remaps the detected lanes coordinate in the image to the actual real-world roads. In this section, we describe a camera calibration method to estimate the camera’s extrinsic parameters, i.e., the height of the camera and the three rotation angles for the transformation from the camera coordinate system to the world coordinate system by three parallel and equally spaced lane-markings on the ground. These extrinsic parameters are necessary for the following lane departure warning step.

**Figure 2:**
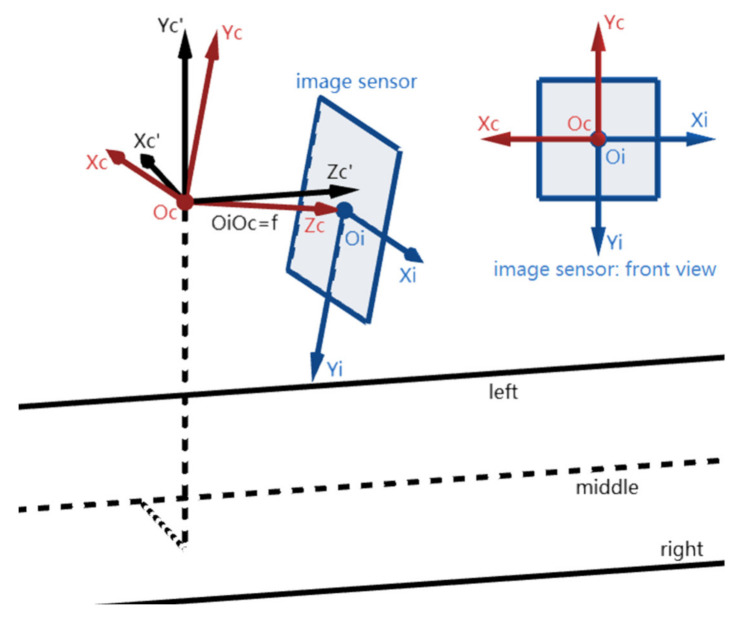
The positions of the three coordinate systems.

Figure 2 shows the positions of the three coordinate systems utilized in this section: (1) the camera coordinate system Oc-XcYcZc; (2) the image coordinate system Oi-XiYi; (3) the world coordinate system Oc’-Xc’Yc’Zc’. Here, Oc-XcYcZc is arbitrary. Oi is at the center of the image sensor (called IMG) where the Zc-axis passes through Oi and is perpendicular to IMG, and f (i.e., the focal length) is the distance between the point Oi and Oc. The Xi- and Yi-axes are parallel to the Xc- and Yc-axes but opposite in direction respectively. Next, the world coordinate system Oc’-Xc’Yc’Zc’ is defined that its origin Oc’ coincides with Oc, its Zc’-axis is parallel to the lane-markings, and its Xc’-axis is parallel to the ground plane.

**Figure 3:**
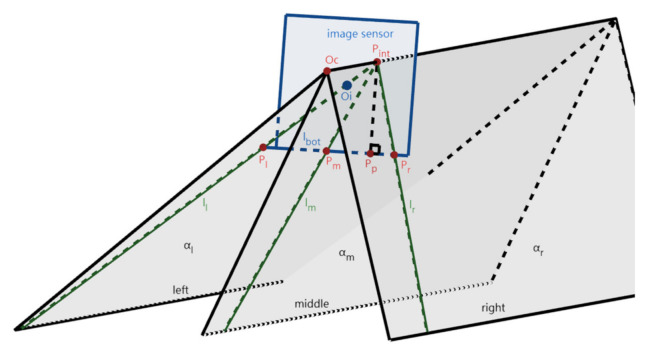
The 3D imaging model of the lane-markings.

Next, the 3D imaging model of the lane-markings is established based on the pinhole camera model, as shown in Figure 3. Each lane-marking and the point Oc determine a plane (α_l_, α_m_, and α_r_), respectively. Here, α_l_, α_m_, and α_r_ intersect the IMG in three lines, i.e., l_l_, l_m_, and l_r_, which are the projections of the left, middle, and right lane-markings onto the IMG plane. l_l_, l_m_, and l_r_ intersect at the point P_int_ (vanishing point of the lane-markings), and l_l_, l_m_, and l_r_ intersect the bottom edge l_bot_) of the IMG at the points P_l_, P_m_, and P_r_. The normal line of l_bot_ at P_int_ intersects l_bot_ at P_p_.

Given the positions of l_l_, l_m_, and l_r_ in the IMG (i.e., the positions of points P_int_, P_l_, P_m_, and P_r_), the coordinate system Oc-XcYcZc is transformed to the Oc’-Xc’Yc’Zc’ following the three steps, (A) rotating θ_1_ around Zc-axis to make Xc-axis on the ZcZc’-plane; (B) rotating θ_2_ around Yc-axis to make Zc-axis coincide with Zc’-axis; (C) rotating θ_3_ around Zc-axis to make Oc-XcYcZc coincide with Oc’-Xc’Yc’Zc’. Meanwhile, the camera height h and then the lane-width w’ are estimated with θ_1_, θ_2_, and θ_3_.

### 2.1. Rotating θ_1_ around Zc-Axis to Make Xc-Axis on the ZcZc’-Plane

**Figure 4:**
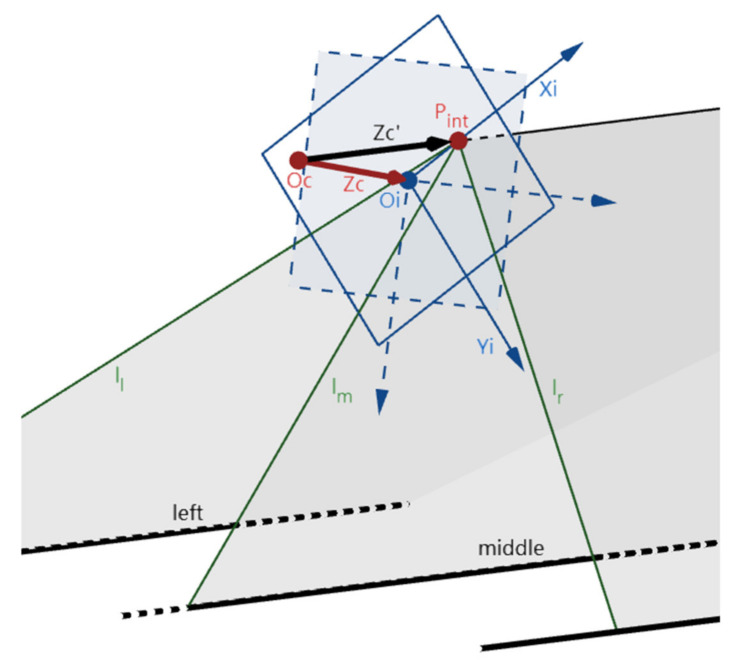
The position change of the IMG in step 2.1. (Xc-, Xc’-, Yc-, and Yc’-axes are omitted).

This step is to rotate an angle around the Zc-axis to make the Xc-axis on the ZcZc’-plane. Afterward, the position of the IMG plane is not changed, but the coordinate system Oi-XiYi is rotated θ_1_ around its origin. Therefore, the positions of l_l_, l_m_, and l_r_ in the world coordinate system remain unchanged while changed in the image coordinate system Oi-XiYi. Because the Xc-axis is on the plane formed by the Zc- and Zc’-axes, P_int_, the intersection of the Zc’-axis and the IMG plane, is still on the Xi-axis as depicted in Figure 4.

**Figure 5:**
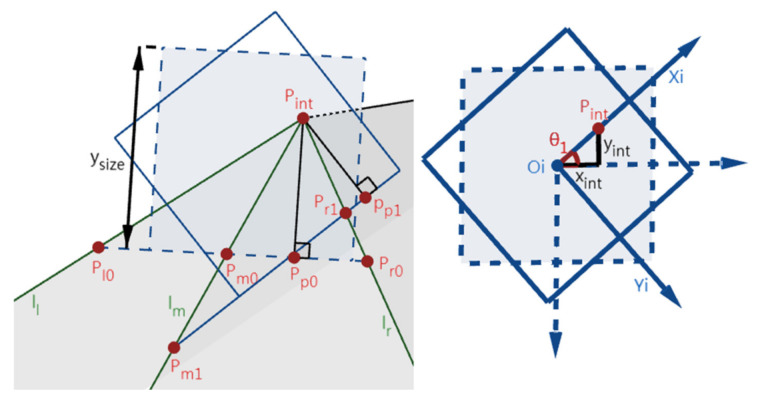
The imaging differences before and after step 2.1.

Figure 5 illustrates the imaging differences before (dash lines, denoted by “0” subscript) and after (solid lines, denoted by “1” subscript) this step. tanθ_1_ can be obtained by dividing y_int_ by x_int_, and the calculated θ1 forms the rotation matrix R_1_ of Oc-XcYcZc in this step. R_1_ is then used in the lane departure warning section (i.e., Section 3, (7)) together with R_2_ and R_3_ calculated later on. P_l1_P_p1_, P_m1_P_p1_, and P_r1_P_p1_ can be obtained by (1) (y_size_ is the height of the IMG).

(1) $$P_{k1}P_{p1}=(\theta_{1}+\arctan\frac{P_{k0}P_{p0}}{P_{\mathrm{int}}P_{p0}})\times \frac{y_{\mathrm{size}}}{2},\;k=l,\;m,\;r$$

### 2.2. Rotating θ_2_ around Yc-Axis to Make Zc-Axis Coincide with Zc’-Axis

**Figure 6:**
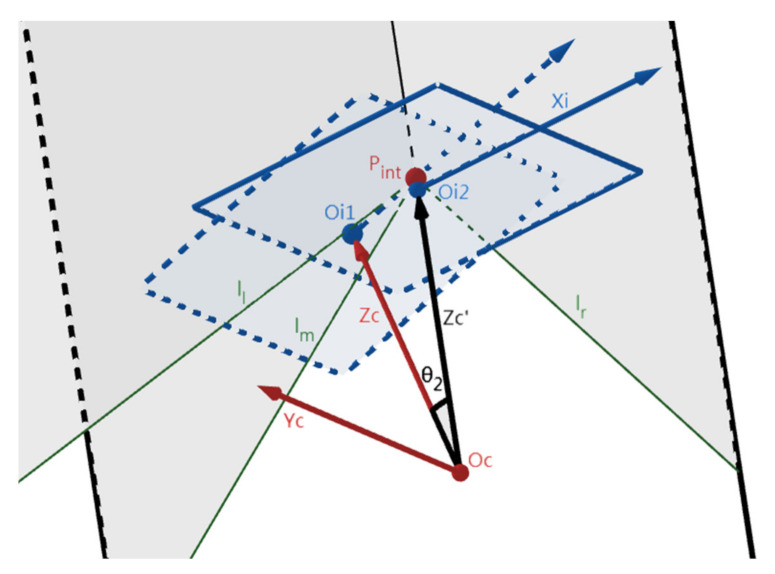
The position change of the IMG in step 2.2. (Xc-, Xc’-, and Yc-axes are omitted).

**Figure 7:**
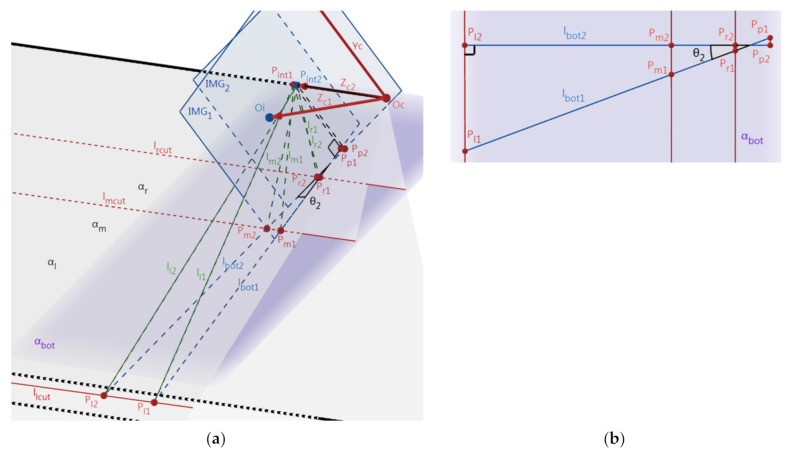
The imaging differences before and after step 2.2. (**a**) The side view of the image sensors. (**b**) The top view of the α_bot_ plane.

This step is to rotate an angle around the Yc-axis to make the Zc-axis coincide with the Zc-axis. Figure 6 depicts the position change of the IMG before (dash lines, O_i1_) and after (solid lines, O_i2_) this step. Then, Zc- and Zc’-axes coincide, while P_int_ coincides with O_i2_. Here, the angle between Zc- and Zc’-axes (i.e., θ_2_) can be calculated with (2) by using the distance between points P_int_ and O_i1_, and the calculated θ2 forms the rotation matrix R2 of Oc-XcYcZc in this step. R_2_ is then used in the lane departure warning section (i.e., Section 3, (7)) together with R_1_ and R_3_ calculated later on. As in Figure 7a, the positions of the IMG planes and lines before and after this step are denoted by the “1” and “2” subscripts, respectively. The two IMG planes are perpendicular to the plane (α_bot_) determined by the bottom lines of the IMG, i.e., l_bot2_ and l_bot1_, and α_l_, α_m_, and α_r_ intersect α_bot_ in three parallel lines (l_lcut_, l_mcut_, and l_rcut_). Lines l_lcut_, l_mcut_, and l_rcut_ intersect l_bot1_ at P_l1_, P_m1_, and P_r1_, and intersect l_bot2_ at P_l2_, P_m2_, and P_r2_. Make two normal lines of l_bot1_ and l_bot2_ at P_int1_ and P_int2,_ respectively, and they intersect l_bot1_ and l_bot2_ at P_p1_ and P_p2_. Because l_bot1_ and l_bot2_ are perpendicular to the Yc-axis, their angle is θ_2_. Meanwhile, because IMG_2_ is perpendicular to the Zc_2_- (which is also Zc’-) axis, l_bot2_ is perpendicular to the Zc’-axis and also l_lcut_, l_mcut_, and l_rcut_. As shown in Figure 7b and Equation (3), using similar triangles, the segments P_l1_P_p1_, P_m1_P_p1_, and P_r1_P_p1_ time cosθ_2_ equals P_l2_P_p2_ P_m2_P_p2_, and P_r2_P_p2_ respectively.

(2) $$\tan\theta_{2}=\frac{O_{i1}P_{\mathrm{int}}}{O_{i1}O_{c}}=\frac{\sqrt{x_{\mathrm{int}}^{2}+\;y_{\mathrm{int}}^{2}}}{f}$$

(3) $$P_{k2}P_{p2}=\cos\theta_{2}\times P_{k1}P_{p1},\;k=l,\;m,\;r.$$

### 2.3. Rotating θ_3_ around Zc-Axis to Make Oc-XcYcZc Coincide with Oc’-Xc’Yc’Zc’

**Figure 8:**
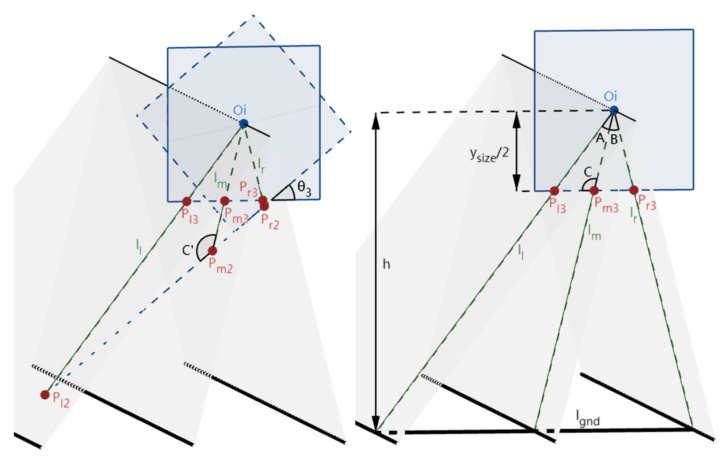
The imaging differences before and after step 2.3.

This step is to rotate an angle around Zc-axis to make Oc-XcYcZc coincide with Oc’-Xc’Yc’Zc’. As illustrated in Figure 8, we denote the positions of the IMG planes and parallel lines before and after rotating θ_3_ around Zc-axis by “2” and “3” subscripts, respectively. Similar to step 2.1, the positions of l_l_, l_m_, and l_r_ in the world coordinate system Oc’-Xc’Yc’Zc’ remains unchanged while their positions in the image coordinate system Oi-XiYi are changed. On the other hand, differing from step 2.1, the position of P_int_ keeps the same (i.e., coincides with Oi) during this step. Because l_bot3_ is parallel to the ground plane, the line of intersection (l_gnd_) of the IMG plane and the ground are also parallel to l_bot3_. From the known condition that the lane-markings are parallel and equally spaced, the two segments cut by the three-lane-markings are equal. By using similar triangles, P_l3_P_m3_ and P_r3_P_m3_ cut by l_l_, l_m_, and l_r_ are also equal. Accordingly, the calculation of θ_3_ is obtained by (4), and the calculated θ3 forms the rotation matrix R3 of Oc-XcYcZc in this step. R_3_ is then used in the lane departure warning section (i.e., Section 3, (7)) together with R_1_ and R_2_. In the previous steps, the calculation of θ_1_ and θ_2_ involved only the positional relationships of points P_int_ and Oi, and only two lane-markings can determine the position of Pint. However, the intersection points of l_l_, l_m_, l_r_, and l_bot_, i.e., P_l3_, P_m3_, and P_r3,_ are needed for the calculation of θ_3_, which demonstrates that three instead of two lane-markings are necessary in the camera-calibration stage.

(4) $$\theta_{3}=∠C-∠C^{\prime }=\arctan\frac{2\sin A\sin B}{\sin\left(A-B\right)}-∠C^{\prime }.$$

### 2.4. Calculation of Camera Height and Lane-Width

**Figure 9:**
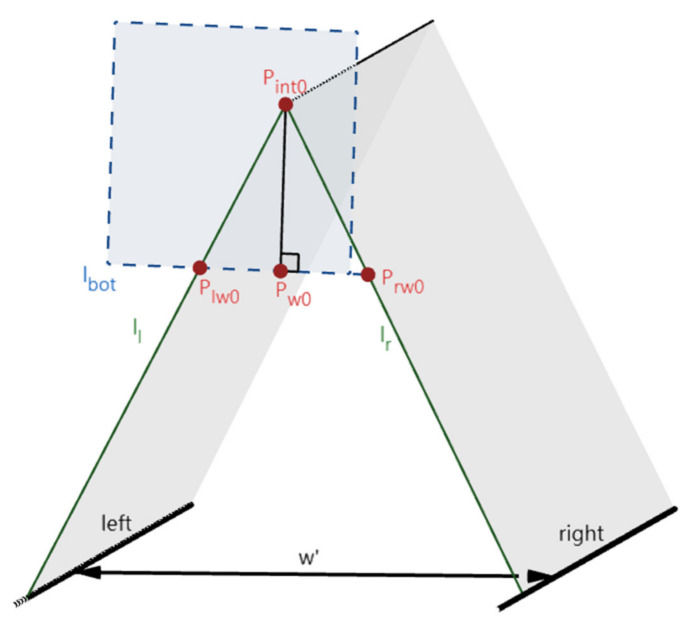
The imaging for the calculation of w’.

After the above steps, the camera height h, the height of Oc, can be calculated according to similar triangles (in (5), w is the distance between the two adjacent lane-markings of the three parallel and equally spaced lane-markings). Since the three-camera rotation angles and the camera height are the known extrinsic parameters, the lane-width w’ can be calculated by both left and right lane-markings, with the vehicle’s direction aligning with the lane-markings. In the case of two edges of the lane-markings, suppose the two lane-markings in the frame intersect l_bot_ at the points P_lw0_ and P_rw0_, and the normal line of l_bot_ at the intersection point (P_int0_) of the two lane-markings intersects l_bot_ at P_pw0_, then w’ is calculated in (6) (shown in Figure 9).

(5) $$\frac{\frac{y_{\mathrm{size}}}{2}}{h}=\frac{P_{l3}P_{r3}}{w}=\frac{y_{\mathrm{size}}}{2w}\times \;[\tan\left(\theta_{3}+\arctan\frac{P_{l2}P_{p2}}{O_{i}P_{p2}}\right)-\tan\left(\theta_{3}+\arctan\frac{P_{r2}P_{p2}}{O_{i}P_{p2}}\right)]$$

(6) $$\left\{\begin{array}{c} P_{\mathrm{lwi}}P_{\mathrm{rwi}}=\frac{y_{\mathrm{size}}}{2}\times [\tan\left(\theta_{i}+\arctan\frac{P_{\mathrm{lw}\left(i-1\right)}P_{\mathrm{pw}\left(i-1\right)}}{P_{\mathrm{int}\left(i-1\right)}P_{\mathrm{pw}\left(i-1\right)}}\right)\\ -\tan\left(\theta_{i}+\arctan\frac{P_{\mathrm{rw}\left(i-1\right)}P_{\mathrm{pw}\left(i-1\right)}}{P_{\mathrm{int}\left(i-1\right)}P_{\mathrm{pw}\left(i-1\right)}}\right)],\;i=1,\;3\\ P_{\mathrm{lw}2}P_{\mathrm{rw}2}=\cos\theta_{2}\times \;\left(P_{\mathrm{lw}1}P_{\mathrm{pw}1}-P_{\mathrm{rw}1}P_{\mathrm{pw}1}\right)\\ \frac{\frac{y_{\mathrm{size}}}{2}}{h}=\frac{P_{\mathrm{lw}3}P_{\mathrm{rw}3}}{w\prime } \end{array}\right.$$

## 3. Lane Departure Warning

The extrinsic parameters, i.e., the rotation angles of the camera coordinate system and the camera height, deduced during the camera-calibration stage, are used to calculate the lane departure parameters. In this paper, the yaw angle (θ_y_), which represents the vehicle direction that deviates from the road direction, can be calculated by only one of the two lane-markings projected in the IMG plane. Meanwhile, the distance between the lane-markings and the vehicle (x_x_) is also important for the lane departure decision. As long as at least one lane-marking is detected in the image by the lane detection technique, the two parameters related to lane departure, θ_y_ and x_x_, can be calculated in this section for lane departure warning.

**Figure 10:**
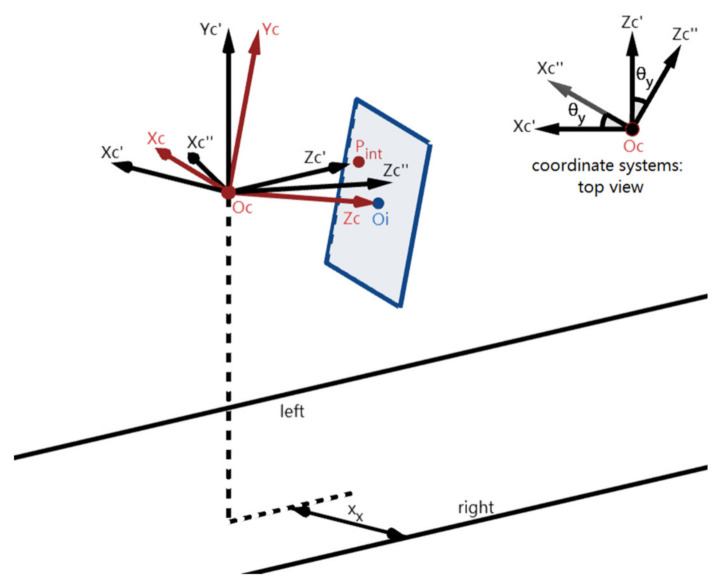
The side and top view of the coordinate systems.

The 3D imaging model of the lane-markings and the coordinate systems Oc’-Xc’Yc’Zc’ and Oc-XcYcZc have been described in Section 2. As illustrated in Figure 10, the vehicle coordinate system Oc’’-Xc’’Yc’’Zc’’ is defined by rotating Oc’-Xc’Yc’Zc’ around the Yc’-axis to make the Zc’’-axis align with the direction of the vehicle. It is observed that the angle between the Zc’’- and Zc’-axis is the yaw angle θ_y_.

### 3.1. Calculation of the Yaw Angle θ_y_

**Figure 11:**
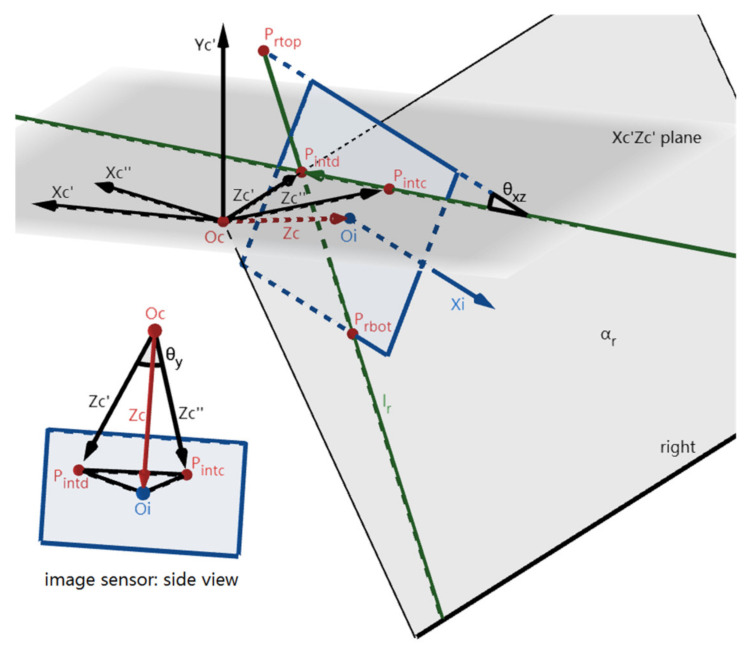
The imaging for the calculation of θ_y_. The right lane l_r_ is detected in the IMG plane.

The first step of lane departure warning is to use only one detected lane-marking in the image to calculate the yaw angle θ_y_, then the angle between the Zc’’- and Zc’-axes can be calculated by finding out the coordinates of intersection points of the IMG and the Zc’’- and Zc’-axis respectively (shown in Figure 11). We define the intersection point of the IMG and the Zc’’-axis as P_intc_(x_intc_, y_intc_), which is the same as P_int_(x_int_, y_int_) in Section 2. Then, the intersection point of the IMG and the Zc’-axis is defined as P_intd_(x_intd_, y_intd_), which is the same as the intersection point of l_l_ and l_r_ in the case of two lane-markings detected. Once only one of the two lane-markings is detected (for example, in Figure 11, l_r_ is detected, and it intersects the top and the bottom edge of the IMG at two points, P_rtop_ and P_rbot_, whose x-coordinates in Oi-XiYi are x_rtop_ and x_rbot,_ respectively), the position of P_intd_ cannot be determined directly. However, it can be calculated using the extrinsic parameters obtained in Section 2.

Since Oc’’-Xc’’Yc’’Zc’’ is defined by rotating Oc’-Xc’Yc’Zc’ around the Yc’-axis, the Zc’’-axis is in the Xc’Zc’-plane and the line P_intc_P_intd_ is the line of intersection of the IMG plane and the Xc’Zc’-plane. Therefore, the angle θ_xz_ between the unit vector **p_int0_** of the line P_intc_P_intd_ and the unit vector **x_i0_** of the Xi-axis can be calculated as shown in (7), using the rotation matrices **R_1_**, **R_2_**, and **R_3_** calculated in Section 2 when **p_int0_** is perpendicular to the unit vector **z_c0_** of the Zc-axis. Then, P_intd_ can be calculated as the intersection point of the detected l_l_ or l_r_ and the line P_intc_P_intd_ as in (8).

(7) $$\left\{\begin{array}{c} \vec{p_{int0}}=\;\left[\begin{array}{ccc} 0 & 0 & -1\\ 0 & 0 & 0\\ 1 & 0 & 0 \end{array}\right]\times \;\vec{z_{c0}}=\left[\begin{array}{ccc} 0 & 0 & -1\\ 0 & 0 & 0\\ 1 & 0 & 0 \end{array}\right]R_{3}R_{2}R_{1}\left[\begin{array}{c} 0\\ 0\\ 1 \end{array}\right]\\ \vec{x_{i0}}=R_{3}R_{2}R_{1}\left[\begin{array}{c} -1\\ 0\\ 0 \end{array}\right]\\ \cos\theta_{\mathrm{xz}}=\cos<\vec{p_{int0}},\;\vec{x_{i0}}>=\frac{\vec{p_{int0}}\;\cdot \;\vec{x_{i0}}}{\left|\vec{p_{int0}}\right|\;\cdot \;\left|\vec{x_{i0}}\right|}=\vec{p_{int0}}\cdot \vec{x_{i0}} \end{array}\right.$$

(8) $$\left\{\begin{array}{c} \frac{y_{\mathrm{intd}}-y_{\mathrm{intc}}}{x_{\mathrm{intd}}-x_{\mathrm{intc}}}=\tan\theta_{\mathrm{xz}}\\ \frac{x_{\mathrm{rtop}}-x_{\mathrm{intd}}}{-\frac{y_{\mathrm{size}}}{2}-y_{\mathrm{intd}}}=\frac{x_{\mathrm{rbot}}-x_{\mathrm{intd}}}{\frac{y_{\mathrm{size}}}{2}-y_{\mathrm{intd}}} \end{array}\right.\Rightarrow P_{\mathrm{intd}}(x_{\mathrm{intd}},{\;y}_{\mathrm{intd}})$$

Finally, with focal length f and the coordinates of points P_intc_, P_intd_, θ_y_ can be solved using the triangular pyramid formed by the axes and the image sensor in Figure 11 according to (9), and the rotation matrix **R_y_** between coordinate systems Oc’’-Xc’’Yc’’Zc’’ and Oc’-Xc’Yc’Zc’ is formed by the calculated θ_y_. **R_y_** is then used in the calculation of x_x_ in Section 3.2, (10). The vectors **OcOi**, **OcP_intd_**, and **OcP_intc_** are called vectors **z_c_**, **z_c’_**, and **z_c’’_** respectively.

(9) $$\left\{\begin{array}{c} \vec{z_{c\prime }}=\vec{z_{c}}+\;\left[\begin{array}{ccc} x_{\mathrm{intd}} & y_{\mathrm{intd}} & 0 \end{array}\right]\;=\;\left[\begin{array}{ccc} x_{\mathrm{intd}} & y_{\mathrm{intd}} & f \end{array}\right]\\ \vec{z_{c\prime \prime }}=\vec{z_{c}}+\;\left[\begin{array}{ccc} x_{\mathrm{intc}} & y_{\mathrm{intc}} & 0 \end{array}\right]\;=\;\left[\begin{array}{ccc} x_{\mathrm{intc}} & y_{\mathrm{intc}} & f \end{array}\right]\\ \cos\theta_{y}=\cos<\vec{z_{c\prime }},\;\vec{z_{c\prime \prime }}>=\frac{\vec{z_{c\prime }}\;\cdot \;\vec{z_{c\prime \prime }}}{\left|\vec{z_{c\prime }}\right|\;\cdot \;\left|\vec{z_{c\prime \prime }}\right|} \end{array}\right.$$

### 3.2. Calculation of the Distance between the Lane-Markings and the Vehicle x_x_

**Figure 12:**
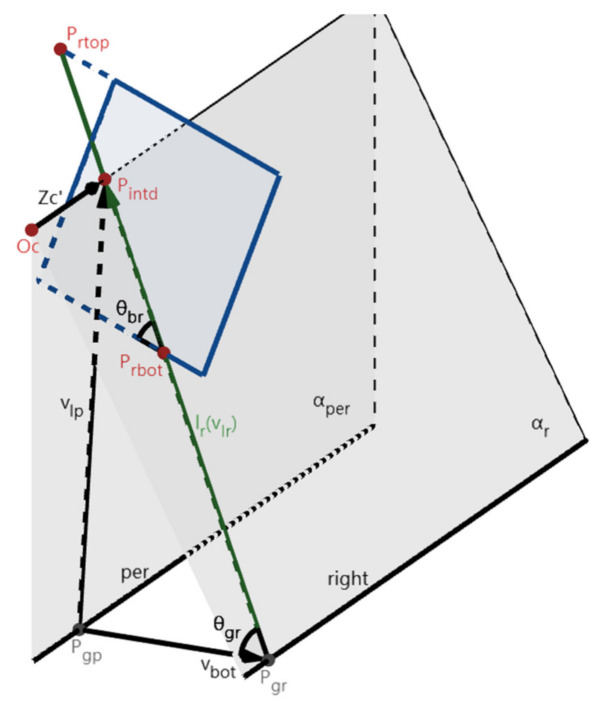
The imaging for the calculation of x_x_. The right lane l_r_ is detected in the IMG plane.

In this paper, x_x_ is calculated using the 3D imaging model of one of the two lane-markings. As an example, in Figure 12, the right lane l_r_ is detected in the IMG plane. Make a plane α_per_ perpendicular to the ground plane through the Zc’-axis, which intersects the ground plane in the line l_per_. It is observed that x_x_ is the distance between l_per_ and the right lane-marking. The IMG plane intersects the lines l_per_ and the right lane-marking at two points P_gp_ and P_gr_. Make vectors **v_bot_**, **v_lp_**, and **v_lr_**, which are vectors **P_gp_P_gr_**, **P_gp_P_intd_**, and **P_gr_P_intd_** respectively. Accordingly, x_x_ is the x-coordinate of **v_bot_** in Oc’-Xc’Yc’Zc’. The angle between **v_lr_** and the bottom edge of the IMG is θ_br_, and the angle between **v_lr_** and **v_bot_** is θ_gr_. Finally, tanθ_br_ can be obtained using the coordinates of points P_rbot_ and P_intd_, and x_x_ can be calculated by θ_br_ through (10). Here, θ_gr_ is calculated by using θ_br_ and θ_xz_ in step 3.1 for the calculation of the yaw angle θ_y_. In particular, **v_bot_** and **v_lp_** can be calculated by using the transformation from Oc’-Xc’Yc’Zc’ to Oc-XcYcZc and the dot product of **v_bot_** and **v_lr_**.

(10) $$\left\{\begin{array}{c} \vec{v_{bot}}=\left[\begin{array}{ccc} 0 & 0 & 1\\ 0 & 0 & 0\\ -1 & 0 & 0 \end{array}\right]\times \frac{x_{x}\;\times \;R_{y}\;\times \;\vec{z_{c0}}}{R_{y}\;\times \;\vec{z_{c0}}\;\times \;\left[0\;0\;1\right]}\\ \vec{v_{lp}}=\left[\begin{array}{ccc} 0 & 0 & 0\\ 0 & 0 & 1\\ 0 & -1 & 0 \end{array}\right]\times \frac{h\;\times \;R_{y}\;\times \;\vec{z_{c0}}}{R_{y}\;\times \;\vec{z_{c0}}\;\times \;\left[0\;0\;1\right]}\\ \vec{v_{lr}}\;\cdot \;\vec{v_{bot}}=\;\left|\vec{v_{lr}}\right|\;\cdot \;\left|\vec{v_{bot}}\right|\;\cdot \;\cos\theta_{\mathrm{gr}}=\;\left|\vec{v_{lp}}-\vec{v_{bot}}\right|\;\cdot \;\left|\vec{v_{bot}}\right|\;\cdot \;\cos\left(\theta_{\mathrm{br}}-\theta_{\mathrm{xz}}\right) \end{array}\right.\Rightarrow x_{x}$$

### 3.3. Lane Departure Assessment

For the real-world application, the departure status of the vehicle is assessed according to the calculated θ_y_ and x_x_. If x_x_ becomes less than a threshold value, the vehicle is approaching the detected lane-marking. If θ_y_ becomes less than a threshold value, it means the vehicle is turning toward the detected lane-marking. These two parameters can efficiently and correctly determine the departure status of the vehicle.

Moreover, the vehicle position relative to the other undetected lane-marking can also be obtained with the calculated lane-width w’. The lane width generally depends on the assumed maximum vehicle width with an additional space to allow for the vehicle motion. In the case of only one edge of the lane, the other edge can also be estimated by a typical lane-width w’’. Therefore, the lane departure is easily determined using the Time to Lane Crossing (TLC) criterion or other criteria. Besides roads with lane-markings, when the vehicle is on the road without any lane-markings, the above lane departure warning method can be used to keep the vehicle in one of the leftmost or rightmost edges of the road.

### 3.4. Lane Detection

As mentioned above, at least one lane-marking should be detected in the image in order to calculate parameters θ_y_ and x_x_. We applied an open-source method proposed by Qin et al. [29] for the lane detection method used in our study. This method is based on deep segmentation, including a novel lane detection formulation aiming at breakneck speed and no-visual-clue problem. The formulation is proposed to select locations of lanes at predefined rows of the image using global features instead of segmenting every pixel of lanes based on a local receptive field, which significantly reduces the computational cost. Previous experiments show that this method could achieve state-of-the-art performance in terms of both speed and accuracy.

## 4. Experimental Results

**Figure 13:**
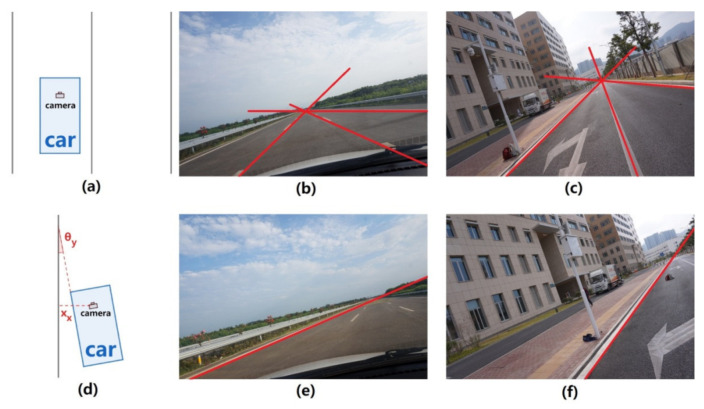
Example frames of the experiments and the corresponding top views of the environments. (**a**) Top view of the camera calibration experiments. (**b**) Camera calibration of the highway experiments. (**c**) Camera calibration of the urban road experiments. (**d**) Top view of the lane departure assessment experiments. (**e**) Lane departure assessment of the highway experiments. (**f**) Lane departure assessment of the urban road experiments.

Experiments were conducted at both highways and urban roads, using image sequences captured by a camera mounted on a car with an arbitrary position. At the beginning of the experiments, the camera is calibrated by parallel placing the car to the lane-markings (i.e., the angle between the car direction and the road direction is zero) while all three lane-markings were in the viewfinder of the camera. The reason for placing the car parallel to the lane-markings is that the car direction when taking the calibration image is an object of reference for real driving, and if it fails to be parallel to the lane-marking, the error in the estimation of θ_y_ becomes large. To avoid the influence of artificial error, we took several calibration images to optimize the parameters. After that, the lane-markings are detected by the lane detection technique. Then, θ_1_, θ_2_, θ_3_, and camera height are calculated as mentioned in Section 2. Figure 13a–c shows example frames for camera calibration and the corresponding top view of the experimental environment of highway and urban road experiments, respectively.

After the calibration step, the position and orientation of the experimental car were arbitrarily changed to simulate the real driving situation while the pose of the camera (i.e., camera coordinate system) relative to the car keeps stable. Then, an image (called “driving image”) was taken and a steel tape manually measured two parameters: (a) the distance from the camera to one of the lane-markings (x_x_); (b) the yaw angle of the experimental car to the lane (θ_y_). The lane detection technique is used again to detect the clearest lane-marking in the “driving image”, and the two-lane departure parameters, i.e., θ_y_ and x_x_, are estimated using the previously mentioned lane departure warning method. Figure 13d–f shows example frames for lane departure assessment and the corresponding top view of the experimental environment of highway and urban road experiments, respectively. To test the algorithm in different situations, the camera’s pose was arbitrarily changed five times in the highway experiment and four times in the urban road experiment. Finally, the estimated quantities and the actual measured values were compared, and the errors were calculated.

**Figure 14:**
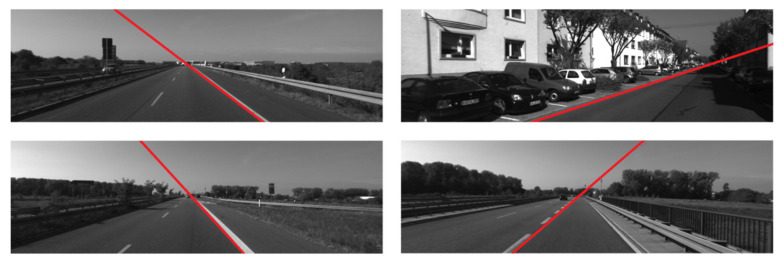
Example frames of the KITTI odometry dataset.

KITTI odometry dataset was initially created for visual odometry or SLAM algorithms [30]. It is almost the only benchmark dataset with ground truth in its NO.00-11 image sequences, including the camera coordinate of each image gathered by a GPS/IMU system. According to the transformation matrix of each image, the vehicle’s deviation angle θ_y_ of each image can be deduced. However, the KITTI does not provide the real values of the distances from the camera to the lane-markings, which impossibly assesses the parameter x_x_. KITTI can objectively quantify the performance analysis of the proposed algorithm and the state-of-the-art works without manually measuring the errors. Figure 14 shows example frames of the KITTI odometry dataset.

Table 1 tabulates the experimental results for lane departure assessment. It is observed that the average error of θ_y_ is about 1 degree, and the average error of x_x_ is less than 5 cm. Causes of errors probably include the small deviation of the vehicle orientation during the calibration process and the measurement errors of real values of the camera positions. To directly evaluate the effect of the warning algorithm, lane departure criteria on both θ_y_ and x_x_ were defined to calculate the correct warning rate. For the highway experiment, the distance from the car’s front wheel to the lane-marking replaces x_x_ as a criterion since this parameter is more direct for departure judgment. For the KITTI dataset experiment, because x_x_ cannot be estimated, only θ_y_ is the criterion.

Table 2 compares this work to six state-of-the-art algorithms where their previous experiments are mainly conducted on their dedicated datasets but not on a public dataset. As in this work, these six algorithms provided their formulas, respectively. This makes the algorithms available to be re-implemented on other datasets. Since these six algorithms only need the expressions of the detected two lane-markings in the images, therefore, the dataset should contain information on at least two lane-markings. Another condition is setting the threshold values in each algorithm while few algorithms give their threshold values. We take the threshold values during the experiments and software-based simulations, resulting in the best correct warning rate.

Finally, only 604 in 1546 frames include two lane-markings in our dataset. The best performance on our dataset among all six is [15], which failed to reach the 90% correct warning rate. The main reason is that these algorithms need two lane-markings while the angles between the two detected lane-marking lines might change drastically with the deviation angles of the camera. For example, the angle bisector of the detected two lane-markings, the parameter of lane-departure judgment in [18], is mainly affected by camera rotation around Zc-axis.

On the other hand, the proposed algorithm is compared with [25], which uses the 3D imaging model to calculate the θ_y_ and x_x_ parameters. The comparison result is shown in Table 3, indicating the performances of the algorithms are both excellent and almost the same. High accuracy indicates the advantage of combining the image information and the road model.

**Figure 15:**
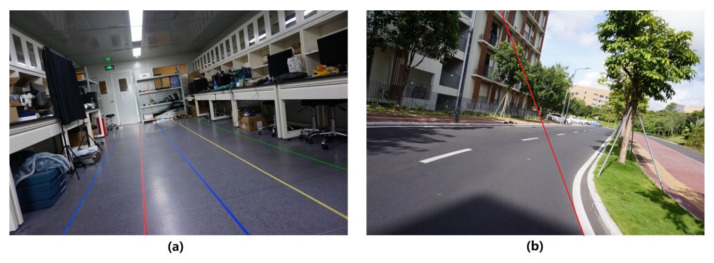
Example frames of the h and w’ experiment (**a**) and the curved road experiment (**b**).

Other than the decision-making parameters θ_y_ and x_x_, the accuracy of camera height h and lane-width w’ is also important. We experimented with camera height and lane width in the laboratory, and the result is shown in Table 4. The total frames for testing h and w’ are 205 and 780, and the average errors are less than 2% and 3%, respectively. Figure 15a shows example frames of the h and w’ experiment.

**Figure 16:**
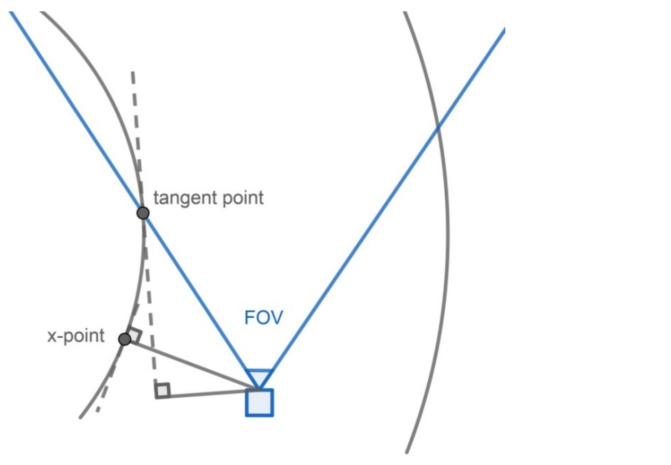
The position of x-point.

As for the curved roads, the tangent line of a curve plays the same role as the “straight lane-marking.” Therefore, the parameter θ_y_ is the vehicle direction that deviates from the tangent line of the curved lane-marking, and the parameter x_x_ is the distance between the vehicle and the tangent line of the curved lane-marking. The experiment for curved roads is carried out to estimate the parameter x_x_, while θ_y_ is not estimated because the direction of the tangent line changes as the vehicle moves, and it is hard to measure its real value. In Table 5, the error of x_x_ is 17.29 cm, and the correct warning rate is 89.25%. The difficulty in detecting the tangent line may cause an error increase, but the main reason is the camera’s field of view (FOV), which causes the difference between the tangent point and the point for measuring x_x_ (called the x-point), as shown in Figure 16. x-point is the closest point on the lane-marking to the vehicle, so the distance from the vehicle to the x-point is the real value of x_x_. However, for the precise detection of the lane-markings in front of the vehicle, the camera should face forward, so the camera usually cannot capture x-point, and the point nearest to the x-point which the camera can capture is tangent-point. Therefore, the road’s curvature causes an error between tangent-point and x-point. The greater the curvature of the lane-marking is, the more significant the difference between the slopes of the tangent lines at tangent-point and x-point is, the bigger the error of the calculated x_x_ is. This error can be possibly reduced by estimating the position of the x-point with advanced algorithms in future research. It may be more natural to detect the curved lane-markings and use the detected curve in the image but not the tangent line to determine lane departure. However, it will be much more complex to calculate the projective relation between a curve in the world coordinate system and that in the image coordinate system. Therefore, using a tangent line is the best solution for a curved lane departure warning. Figure 15b shows example frames of the curved road experiment.

## 5. Conclusions

This paper proposed a lane departure assessment method with precise positioning through a 3D camera imaging model. We exhibited the advantages of this method in three aspects. First, the calibration environment is simple to be equipped with no requirements for camera installation. The camera can be arbitrarily installed in the vehicle, and the environment only needs to contain three parallel and equally spaced horizontal lines. Second, the camera height is calibrated to avoid measurement difficulty and errors. The camera focal length, which is relatively constant, is used in calibration to calculate the camera height. Third, the critical parameters of the departure determination, i.e., the yaw angle representing the deviation of vehicle direction and the distance between the lane-marking and the vehicle, can be deduced by even only one lane line, which is valuable and reliable to the real-world applications. Finally, the experiment results illustrated the high accuracy of the lane departure assessment. The drawback of the proposed method lies in the curved lane departure warning, which requires a high camera’s field of view for low estimated error. The proposed algorithm can improve traffic safety and has excellent potential to be applied to future intelligent transportation systems.

## Figures and Tables

**Table 1:** The experimental results for lane departure assessment.

Experiment	Tested Frames	θ_y_ Error	x_x_ Error	Departure Frames	False Alarms	Correct Warning Rate	Lane Departure Criteria	Image Resolution	Camera Height
Highway	109	0.36°	4.24 cm	27	2	98.17%	x_fw_ * < 80 cm & θ_y_ ≥ 15°	5456 *×* 3632	120 cm
Urban Road	1546	1.13°	4.64 cm	305	21	98.64%	x_x_ < 150 cm & θ_y_ ≥ 15°	5456 *×* 3632	144 cm
KITTI	533	0.97°	-	20	0	100%	θ_y_ ≥ 15°	1241 *×* 376	165 cm
Sum	2188	1.05°	4.61 cm	352	23	98.95%	-	-	-

* x_fw_ is the distance from the front wheel of the experimental car to the lane-marking.

**Table 2:** Comparison of the proposed algorithm with state-of-the-art algorithms on lane departure warning.

Algorithm	Total Frames	Departure Frames	False Alarms	Correct Warning Rate
Chen and Jiang [18]	604	101	422	30.13%
Petwal and Hota [16]	604	101	299	50.50%
Gamal et al. [19]	604	101	241	60.10%
Bhujbal and Narote [12]	604	101	187	69.04%
Yu et al. [23]	604	101	134	77.81%
Viswanath et al. [15]	604	101	61	89.90%
This Work	1546	305	21	98.64%

**Table 3:** Comparison of the proposed algorithm with [25] on lane departure warning.

Algorithm	Total Frames	θ_y_ Error	x_x_ Error	Departure Frames	False Alarms	Correct Warning Rate
Xu and Wang [25]	1546	1.36°	5.19 cm	305	21	98.64%
This Work	1546	1.13°	4.64 cm	305	21	98.64%

**Table 4:** The experimental results of camera height h and lane-width w’. (Error1 is error in cm and Error2 is error in percentage).

h (cm)	Frames	Error1	Error2	w’ (cm)	Frames	Error1	Error2
75	36	1.39 cm	1.85%				
84	22	1.56 cm	1.86%				
96.6	23	1.61 cm	1.67%	60	312	1.73 cm	2.88%
74.2	29	1.27 cm	1.71%	120	234	2.49 cm	2.08%
84.3	29	1.22 cm	1.45%	180	156	2.91 cm	1.61%
94.6	34	0.87 cm	0.92%	240	78	4.07 cm	1.69%
87.6	32	1.03 cm	1.17%				
Sum	205	1.25 cm	1.50%	Sum	780	2.43 cm	2.27%

**Table 5:** The experimental result of the curved roads.

Section	Frames	x_x_ Error	Departure Frames	False Alarms	Correct Warning Rate
1	43	16.19 cm	16	6	86.05%
2	50	18.23 cm	26	4	92.00%
Sum	93	17.29 cm	42	10	89.25%

## Data Availability

Publicly available datasets were analyzed in this study. This data can be found here: (http://www.cvlibs.net/datasets/kitti/eval_odometry.php) accessed on 26 January 2022. Other data presented in this study are available on request from the corresponding author. The data are not publicly available due to copyright.
